# Sensor Architectures and Technologies for Upper Limb 3D Surface Reconstruction: A Review

**DOI:** 10.3390/s20226584

**Published:** 2020-11-18

**Authors:** Alessandro Paoli, Paolo Neri, Armando V. Razionale, Francesco Tamburrino, Sandro Barone

**Affiliations:** Department of Civil and Industrial Engineering, University of Pisa, Largo L. Lazzarino 1, 56122 Pisa, Italy; a.paoli@ing.unipi.it (A.P.); a.razionale@ing.unipi.it (A.V.R.); francesco.tamburrino@ing.unipi.it (F.T.); sandro.barone@unipi.it (S.B.)

**Keywords:** upper limb 3D scanning, structured light scanning, stationary scanner, handheld scanner, depth cameras, body scanner

## Abstract

3D digital models of the upper limb anatomy represent the starting point for the design process of bespoke devices, such as orthoses and prostheses, which can be modeled on the actual patient’s anatomy by using CAD (Computer Aided Design) tools. The ongoing research on optical scanning methodologies has allowed the development of technologies that allow the surface reconstruction of the upper limb anatomy through procedures characterized by minimum discomfort for the patient. However, the 3D optical scanning of upper limbs is a complex task that requires solving problematic aspects, such as the difficulty of keeping the hand in a stable position and the presence of artefacts due to involuntary movements. Scientific literature, indeed, investigated different approaches in this regard by either integrating commercial devices, to create customized sensor architectures, or by developing innovative 3D acquisition techniques. The present work is aimed at presenting an overview of the state of the art of optical technologies and sensor architectures for the surface acquisition of upper limb anatomies. The review analyzes the working principles at the basis of existing devices and proposes a categorization of the approaches based on handling, pre/post-processing effort, and potentialities in real-time scanning. An in-depth analysis of strengths and weaknesses of the approaches proposed by the research community is also provided to give valuable support in selecting the most appropriate solution for the specific application to be addressed.

## 1. Introduction

The reconstruction of human body parts plays a significant role in many application fields such as human anatomy, medical rehabilitation, apparel industry, ergonomics. In the human anatomy field, for example, body measurements are used to record the evolution of the population [1]. In the apparel industry, body measurements are used to improve body fitting of garments [2,3], while ergonomics adopt measurements to enhance human interaction with the product [4]. In the field of patient rehabilitation, 3D body scanning is considered a crucial starting point for subsequent 3D model design and fabrication of bespoke devices, especially when additive manufacturing techniques are involved in their production [5,6]. The captured 3D digital information can be efficiently used for the design of highly customized medical devices, such as orthoses or controllers, which perfectly fits the patient’s anatomy, thus enabling more comfortable and effective therapies. In this context, the rehabilitation of upper limb mobility takes advantage of using these devices to navigate virtual environments, thus increasing the accessibility of virtual reality (VR) technology also for patients affected by physical disabilities. Upper limb rehabilitation involves working with patients who suffer from pain and/or inhibited function of the musculoskeletal system, which could be due to sports injuries, strokes, or dystonia disorders. Rehabilitation for athletes, serious amateurs, or recreational users is aimed at recovering health and fitness and to alleviate symptoms using rehabilitative exercise, movement, and manual therapeutic intervention. Rehabilitation following strokes refers to the systematic training of muscle groups to re-establish bodily control. Patients affected by hyperkinetic movement disorders, caused by cerebral palsy, Huntington’s disease, or Parkinson’s disease, would benefit from the use of physical user interfaces that analyze involuntary movements and help to attain their control, also inferring intentions from real-time data, thus increasing social and physical participation in meaningful activities. The development of bespoke assistive controllers requires an advanced biomechanical profile of the patient. For this reason, a data acquisition phase is needed to measure 3D surface anatomical data, range of motion and musculoskeletal strength. The 3D scanning of the patient’s upper body anatomy represents the starting point for the creation of the patient’s biomechanical profile. Traditionally, hand and wrist anthropometrics is carried out by contact measurements using calipers. Goniometers may also be used to assess joint range of motion [7]. These procedures, however, are intrusive and time-consuming. Additionally, accuracy is a major concern since manual measurements are characterized by a high degree of subjectivity depending on the operator who carries out the measurement process [8].

**Figure 1:**
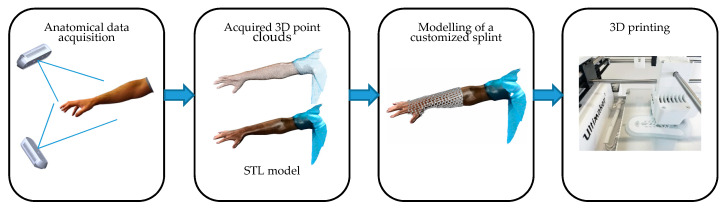
Pipeline illustrating the main steps for the creation of a bespoke splint.

In the last few decades, 3D optical scanning systems have progressively replaced manual measurements in the surface acquisition of human body parts, shortening the acquisition process and increasing reliability and data accuracy [9]. The resulting digital model of the anatomical shape can be used to extract measurements of interest at any time and to help the design of bespoke devices as orthoses, to either correct posture or movement, prostheses, to substitute a body part while maintaining its functionality, or active controllers for rehabilitation assisted by VR environments. These devices are typically hand-crafted since the patient’s morphology is taken by plaster molds and the custom fabricated prototype is adjusted when worn by the patient to increase fitting and enhance comfort. Therefore, the effectiveness of the final product depends on the specialist’s skills and experience. Recently, this standard procedure has been progressively supported and partially replaced by computer-aided design (CAD) and computer-aided engineering (CAE) tools, which allow the design of medical devices based on 3D virtual models that reproduce the actual patient’s anatomy [10]. A great step forward, however, has been achieved by the advent of rapid prototyping (RP) technologies as, for example, additive manufacturing (AM), which have allowed the direct production of fully functional parts from a 3D model without traditional machining process and/or manual processes [11,12]. Figure 1 shows the typical workflow for the development of bespoke devices, which starts from the 3D scanning of the upper limb anatomy and ends with the 3D printing process.

Capturing the 3D shape and motion of human upper limbs has been studied for decades due to its applications in many engineering fields as computer graphics, animation, human–computer interaction, rehabilitation, and robotics. The recent advancement in the field of consumer RGB-D sensors has further increased this interest, especially for virtual and augmented reality applications [13]. There are many technologies available on the market and countless are the approaches proposed by the scientific community. The present work is aimed at presenting an overview of the state of the art of the existing 3D surface data capture methodologies for human upper limb scanning. The paper focuses on optical scanning, without analyzing computed tomography (CT) and magnetic resonance imaging (MRI). These technologies are not considered suitable for the design of bespoke devices due to their high cost, high invasiveness (i.e., radiation issues for CT), and low equipment availability in all clinical centers [8]. Furthermore, many categories of patients cannot easily use this equipment typology due to their health conditions (e.g., dystonia patients which are affected by involuntary movements). Additionally, only papers referring to the direct scanning of the subject’s anatomical region were considered, discarding all the papers that exploit the acquisition of plaster casts to obtain 3D digital models. Hand pose estimation and tracking, which also represents a wide research area [14,15,16], was not considered in this work. It must be noted that this review aims to cover the devices and architectures that have been used at least once to create a virtual model of the patient’s upper limb anatomy, rather than addressing all the existing papers which use a 3D scanning process for this purpose. Other papers certainly exist on this topic since the design process of bespoke devices represents a wide research area, and a complete list of all the works would have made the work hardly readable and redundant. Thus, a selection was performed to describe the sensor architectures which completely represent the state of the art, thus representing valid support for all those researchers who need to build digital models of upper limbs.

## 2. 3D Scanning Technologies

Many technologies are available to perform 3D surface scanning of human body parts. Each technology has its peculiarities, which affect reliability, accuracy, data consistency, scanning time, and costs. For this reason, in this section, an overview is presented, focusing on techniques at the base of commercial devices and research approaches. A main taxonomy could classify these techniques into three categories [17]: time-of-flight (TOF) techniques, passive image-based techniques, and active image-based techniques (structured light, SL).

### 2.1. Time-of-Flight Techniques

Time-of-flight (TOF) techniques use an active emitter, which projects light signals on the surface, and an optical sensor that collects the reflected light from the object. The 3D information is determined by computing the time delay between the light signal emission and the reflected light signal recording. The time delay can be directly measured or indirectly obtained by analyzing the phase difference between the emitted and recorded light. In the first case, the device measures the time of a laser light pulse to complete a round trip travel from the emitter to the receptor. These devices are known as pulsed-light sensors and are characterized by light emitters with high power. Thus, they can measure objects within a long-distance range (10–1000 mt) and without restrictions introduced by environmental conditions. Indeed, they are often used for outdoor measurements of very large objects, such as buildings, ships, or archeological sites. On the other hand, devices measuring the phase difference between emitted and back received light are known as continuous wave modulated light sensors. They are based on the demodulation of the light signals and require a phase unwrapping algorithm. This makes them more suitable for indoor applications and short distances [18].

TOF systems are usually very compact straightforward 3D measurement techniques, and thus suitable for mobile applications since stereo triangulation is not required to retrieve 3D information [19,20]. The target surface is scanned point-per-point in an array of arbitrarily dense sampling points and a depth map is created. However, the achievable depth resolution cannot be too high for short-range measurements because of the high light speed and the limited temporal resolution of the transmitter-receiver system [18]. Consequently, the measurement error of TOF techniques is usually greater than several millimeters, which is significantly worse than that of many structured light methods and stereo vision methods [21,22,23,24]. However, TOF cameras are portable, cheap, and more robust to the interference of external light than SL methods or stereo vision methods, which is attractive for many practical applications where real-time measurement, but not high accuracy nor high resolution, is required. The most recent and representative TOF devices for 3D reconstruction are the Kinect V2 and Azure Kinect [25]. While the former is widely used for 3D scanning applications, the latter has been launched very recently (March 2020) and up to now has been used for hand gesture recognition [26] or gait analysis [27]. However, to the best of the authors’ knowledge, no scientific papers exist documenting the use of the Azure Kinect to reconstruct upper limb geometry.

An emerging variant of pulsed light TOF cameras is represented by LIDAR technology (which stands either for “Light Imaging Detection And Ranging” or for “LIght and radar”). LIDAR is a remote sensing technology that estimates range (or distance, or depth) by illuminating an object with a collimated laser beam and detecting the reflected light using photodetectors. LIDARs usually are high-resolution devices because they use a fine laser-beam (that can be of ultraviolet, visible, or infrared light) [18]. To obtain a full field of depth values, LIDAR sensors must be moved vertically and horizontally to perform a scan of the scene. This can be obtained by using rotating mirrors, or multiple laser beams and detectors, or by using multiple mirrors at fixed orientations, or by diverging the light coming from a single emitter for illuminating the entire scene of interest, then imaging the reflected light onto a 2D array of photodetectors (namely a TOF depth camera). This technology was recently implemented by Intel in the low-cost device RealSense LiDAR Camera L515 [28], whose use for the data capturing of human anatomy, however, has not yet been documented within the scientific literature.

### 2.2. Passive Image-Based Techniques

These techniques are based on the interaction between light and objects imaged by a camera. The 3D information is extracted by complex image processing algorithms, which can correlate the distance of each image pixel from the imaging device by modeling the variation of grayscale shading (shape-from-shading) [29] or the local degree of blur (shape-from-focus/defocus) [30].

Among image-based techniques, stereo-vision certainly represents one of the most commonly used. At least two cameras, viewing the target object from different perspectives, are used to simulate the human stereoscopic vision. Common features on the images are identified (stereo-matching, e.g., basing on epipolar geometry and disparity maps) and the 3D information is retrieved by using standard triangulation techniques, which require the knowledge of the cameras’ projection centers and their relative distance/orientation (camera calibration) [31].

Image-based techniques require low-cost setups since a single camera, or at most two cameras in the case of stereo-vision setups, are needed. On the other hand, depth accuracy is limited, especially for shape-from-shading and shape-from-focus techniques, and real-time measurements are affected by the robustness and speed of the image processing algorithms. For stereo-vision setups, the challenging issue is to find a robust stereo-matching between two views of the same target surface [32]. For this reason, the accuracy of passive stereo-vision ranges from 0.2% for surfaces with good texture to 9.4% for texture-less surfaces [17]. In some cases, where possible, the surface texture can be enhanced by spraying speckle patterns on the target surface, and stereo digital image correlation techniques are used to find correspondences between left and right images [33,34,35].

Photogrammetry (PG) can be considered as an extension of passive stereo vision when more than two cameras are used. Alternatively, the same device can be used to take multiple photographs of the target surface from different viewpoints. While in this latter case the process can be very time-consuming, the former setup can acquire real-time data by synchronizing the imaging devices. Multidevice systems can be used to simultaneously acquire 2D photographs (or 3D scans) from different viewpoints, which are then aligned in a common reference system using surface texture and/or markers placed on the surface or by exploiting ad-hoc calibration procedures. In the healthcare field, photogrammetry is often used to develop whole-body scanners [36,37,38].

### 2.3. Structured Light-Based Techniques

Light projectors (laser or white light) can be added to the measurement system besides the stereo-vision setup, or by simply replacing one of the two cameras. The light source is used to illuminate the scene with structured light patterns, which are deformed by the target’s surface shape. The projection of known structured patterns allows increasing the robustness in determining the one-to-one correspondence between a projector point and a camera point or between corresponding points of two camera images. This provides a robust solution to the stereo matching problem, high measurement accuracy, and 3D reconstruction can be carried out exploiting stereo-triangulation. Several techniques exploit this principle.

In Moiré profilometry [39,40], two identical but slightly misaligned grids are used to create an interference pattern. The reference grid is projected while the misaligned one is placed between the camera and the object. The resulting interference pattern is used to determine contours having equal height. The experimental setup of Moiré profilometry, however, is burdened by the requirement of having a physical demodulation grid. More recently, many other structured light techniques have been developed using a multimedia projector to illuminate the target surface with single or multiple sequential fringe patterns, allowing the definition of more robust and stable measurement systems. The phase-shifting method, with three or four phase-shifts, is the most commonly adopted methodology to retrieve the phase map. Multiple frequencies can be used to increase the acquisition robustness, and also nonsinusoidal fringe patterns have been studied, such as trapezoidal [41], triangular [42], or binary [43,44,45]. These approaches are limited to the acquisition of dynamic targets with relatively slow motions since multiple patterns must be projected, even if the use of high-speed cameras and projectors can improve the results. One-shot structured light techniques can remove this restriction; they have been demonstrated to be suitable for moving targets. A well-known approach that allows the use of the phase-shifting technique with the projection of a single pattern consists of coding the three phase shifting patterns into the RGB channels of a color camera [46,47].

Fourier transform profilometry (FTP), windowed Fourier transform profilometry, and wavelet transform profilometry are other well-established one-shot techniques for 3D shape measurements [48,49]. Standard FTP typically employs a sinusoidal pattern to cover the entire object surface and a reference plane, thus enabling high-resolution, full-field analysis. However, band-pass filtering in the frequency domain causes the loss of high-frequency components, which contain detailed information of the measured object, providing smoother reconstructions with respect to the actual shapes. Moreover, the acquisition of objects characterized by a texture that varies across the surfaces is challenging since it becomes difficult to separate the average intensity from the carrier frequency. In general, all transform-based profilometry techniques are characterized by poor performances around phase map discontinuities [50].

It is worth noting that the phase-based structured light techniques that use an inverse or an arctangent function in the digital signal processing give phase information, which is constrained by its domain value (i.e., [−𝜋, +𝜋)). These constrained or “wrapped” phases are used to obtain a continuous phase map by phase unwrapping algorithms that remove the 2𝜋 discontinuities (phase jumps). These algorithms must be a trade-off between speed and accuracy, and often represent a real bottleneck for real-time reconstructions [51]. Conventionally, a spatial or temporal phase unwrapping algorithm can be adopted to unwrap the phase map. A spatial phase unwrapping algorithm analyzes the wrapped phase map itself and determines the number of 2𝜋’s (or fringe order) to be added to a point based on surface smoothness assumptions. The temporal phase unwrapping algorithm, in contrast, captures additional images to determine the fringe order uniquely for each pixel. Spatial unwrapping is therefore suitable for objects having a continuous and smooth surface and it could fail when abrupt geometrical variations occur (step height more than 2𝜋). Temporal unwrapping, on the other hand, can handle arbitrary geometries at the expense of measurement speed.

In the field of one-shot techniques, an alternative to phase patterns is represented by patterns consisting of dot arrays [52,53,54,55,56], unidirectional lines [57,58,59], or crossed lines [60,61,62]. Edge detection or segmentation algorithms are required to identify the deformed patterns captured by the cameras. In this context, the use of color-coded patterns can simplify the segmentation processes since the color can be used to uniquely label the projected features [54,59]. However, the results are highly influenced by the differences between projected and imaged color values, which could be significant in the measurement of colored surfaces.

An alternative light coding method consists of projecting a statistically random speckle pattern, which locally encodes unique features [63]. For any given point on the camera image, the corresponding projector point can be uniquely determined. Typically, a rectangular kernel is designed to be unique within the whole projected pattern. This technique is characterized by a simple implementation since only a single statistically random pattern must be generated and projected by either a laser speckle projector or a multimedia projector. On the other hand, there are limitations in the low spatial resolution, high sensitivity to noise, and low measurement accuracy [64]. In particular, the accuracy is influenced by the size of the rectangular kernel used to statistically find the most suitable matching point, rather than a point defined by some mathematical relation. The low accuracy of the correspondence determination brings to low measurement accuracy. The statistically random coding method has been adopted by some consumer products as Microsoft Kinect v1 and Intel RealSense due to the ease of implementation and miniaturization.

Overall, structured-light and stereo-vision techniques have very similar measurement accuracy. It can be estimated between 0.01 and 0.5 mm depending on the working volume, or even 0.001 mm for microscopic applications [17]. For a deeper insight into structured light techniques for real-time 3D shape acquisitions, the reader can refer to [64,65,66].

### 2.4. 3D Scanning Technologies Comparison

The previous sections highlighted the main strengths and weaknesses of the available 3D scanning technologies. The presented data are summarized in Table 1, where the three main technologies are compared in terms of robustness to noise sources (e.g., ambient light) and elaboration ambiguity (e.g., phase unwrapping), spatial resolution, processing of the acquired data (which is not referred to the point cloud elaboration), applicability to real-time application and accuracy (i.e., fidelity of the scanning to ground truth).

As can be noted, robustness is a severe issue for passive image-based technologies, since they must rely on the target object patterns, which do not guarantee optimal results. To this extent, it is worth noting that robustness and processing complexity are not directly related, since the highly robust methods can be either characterized by complex or simple data processing algorithms and vice versa.

On the other hand, structured light methods are characterized by higher robustness, since known patterns are projected on the scene, thus increasing the available information, which simplifies the processing algorithms. Anyway, this method can be subjected to ambient light, thus controlled lighting conditions are usually required. A distinction must be made between single frame and multiple frame approaches: in the single frame methods, the intrinsically poor information available to extract 3D data reflects in more complex process algorithms, which generally guarantee a lower success rate with respect to multiple frames methods. Indeed, some single frame methods rely on the color information to enrich the available data, thus being equivalent to a three-frame acquisition, gaining higher robustness but reducing the available frame rate. The table highlights that multiple frames methods achieve the best performances in terms of robustness, resolution, processing complexity, and accuracy, but they result in long acquisition time, being not suitable for real-time applications (such as body scanning). Just a few exceptions exist, based on optimized hardware to speed up the projection and acquisition of multiple frames, but they are still affected by small movement occurring between the projection of different patterns. Finally, photogrammetry techniques can be applied to real-time applications only if many sensors are simultaneously used to acquire the target surface from many different viewpoints. In this scenario, the acquisition itself can be fast (depending on the adopted cameras), but the subsequent data processing is time-consuming, and generally performed offline. It is worth noting that the accuracy level required for human body reconstruction (≈1 mm) is much lower with respect to mechanical metrology application (<0.1 mm), while the acquisition speed becomes a key feature to avoid artefacts introduced by the subject’s movements.

## 3. 3D Scanner Architectures for the Reconstruction of Upper Limb Anatomy

This section describes the broad range of scanners used in the reconstruction of the hand–wrist anatomy, proposing a classification based on aspects such as handling, pre/post-processing effort, and potentialities in real-time scanning. The review will consider both research approaches and consumer products, describing their strengths and weaknesses.

### 3.1. Stationary Scanners

Stationary scanners are composed of optical elements, arranged to define a stereo-vision configuration. Typically, they are based on a light source and one or two cameras, which must be calibrated to obtain reliable surface measurements. Structured light patterns are projected on the scene and imaged by the camera(s). In [67], a full-resolution, high-speed 3D measurement system has been developed by implementing a multiview phase-shifting technique. The system, which consists of a projector and two cameras, has been experimented in scanning a moving hand, with an acquisition rate of 220 Hz and a resolution of 1024 × 768, with the aim of performing gesture recognition. The use of two cameras provides additional information for the direct determination of corresponding points from the wrapped phase maps without phase unwrapping.

**Figure 2:**
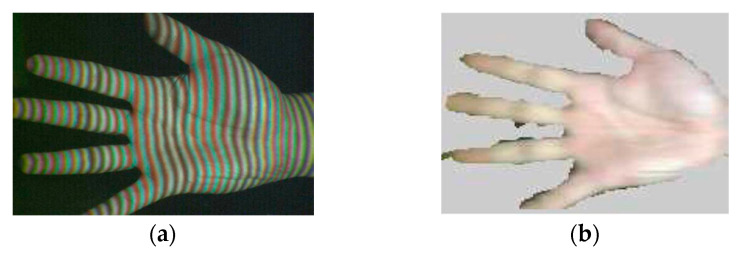
3D hand reconstruction carried out in [68], (**a**) captured image of the hand under composite red, green and blue (RGB) fringe pattern illumination, (**b**) 3D hand model.

A single composite red, green, and blue (RGB) projected fringe pattern is used in [68] and fringe processing is carried out by Fourier transform. The color channels are used as a carrier for fringes at three different frequencies, which are chromatically separated in the primary red, green, and blue channels of the RGB projected image, as shown in Figure 2. The hardware setup, which consists of a DLP video projector and a color camera, has been used to acquire 3D shape and color information of a moving hand. Results evidenced the presence of some noisy data in areas having a significant slope (tips and finger edges, Figure 2b) since Fourier transform profilometry has a limited range of surface slopes that can be correctly determined. Fourier transform profilometry is also used in [69,70], while wavelet transform profilometry is adopted in [71] to reconstruct the hand 3D shape.

**Figure 3:**
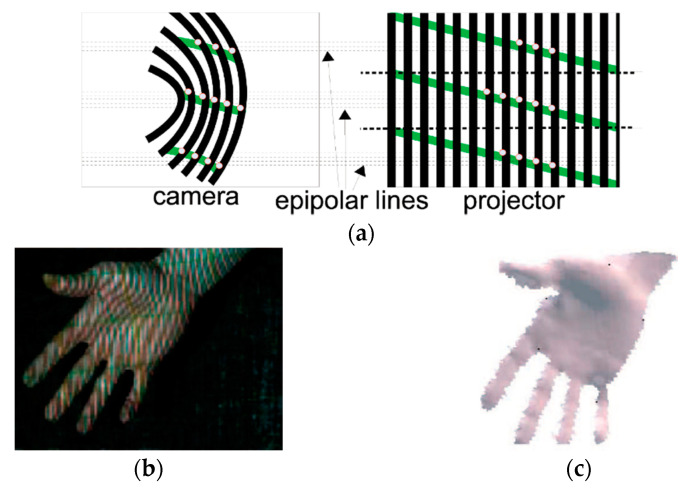
(**a**) Line pattern adopted in [58] along with the geometric coding based on the epipolar constraint, which resolves the ambiguities due to the periodicity of the stripe pattern, (**b**) hand under structured light illumination, (**c**) 3D hand model.

Three unidirectional line patterns techniques have been developed in [58,59,72]. In [58], a single frame pattern composed of black and white fringes is adopted, as shown in Figure 3. The hardware is composed of a consumer-grade projector and camera and the correspondence problem between camera and projector is obtained by a combination of geometric coding, color coding, and tracking over time (Figure 3a). The system has been tested in the reconstruction of a moving hand (Figure 3b). However, problems have been evidenced in the correct code assignment to the thumb and fingers in general, which has turned into an incorrect depth measurement (Figure 3c).

**Figure 4:**
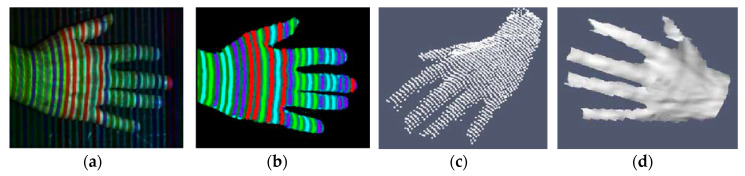
3D hand reconstruction carried out in [59], (**a**) captured image of the hand under pattern illumination, (**b**) color segmentation of the image, (**c**) point cloud, (**d**) 3D hand model.

In [59], a one-shot technique is used by exploiting colored stripe patterns (optimized De Bruijn sequence) to reconstruct a human hand, as shown in Figure 4. The system is composed of a color camera and a digital projector. The decoding process is based on horizontal scan lines and since only four colors are projected (Figure 4a), the color of the detected slits can be easily segmented (Figure 4b). The skin usually introduces a strong gain on the red component of the pattern and, at the same time, attenuates the overall luminosity reaching the camera. However, the proposed technique allows reconstructing fine details of the hand such as veins (Figure 4c,d).

**Figure 5:**
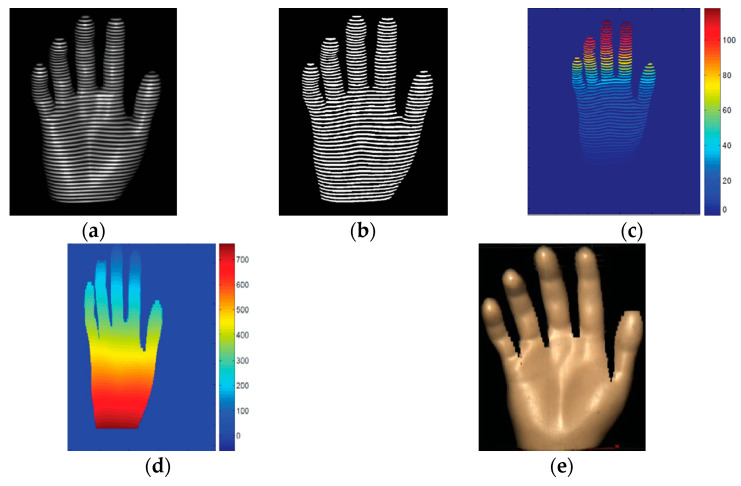
3D hand reconstruction carried out in [72], (**a**) captured image, (**b**) segmentation process, (**c**) line clustering, (**d**) coordinate shift map, (**e**) 3D hand model.

In [72], a single-shot 3D hand reconstruction approach based on the structured light line pattern is proposed (Figure 5). A projector is used to project a set of bright parallel straight lines on the target surface. A camera acquires the lines distorted by the surface (Figure 5a) and performs segmentation (Figure 5b) and clustering processes (Figure 5c), based on the hand features. The line coordinate shift map, which represents the distortion of the projected straight lines, can then be determined (Figure 5d). The developed system proved to be promising for real-time applications, such as hand reconstruction (Figure 5e), with an accuracy of 0.2%. 

**Figure 6:**
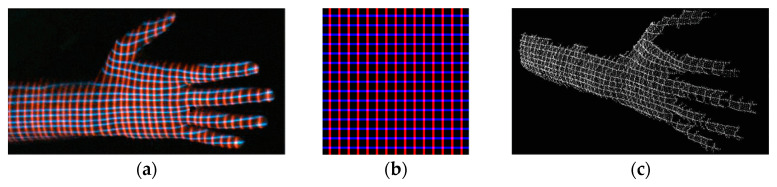
3D hand reconstruction carried out in [62], (**a**) captured image (**b**) projected pattern, (**c**) reconstructed 3D points.

In [62], a one-shot technique based on the projection of crossed lines forming a spaced grid is presented. Both vertical and horizontal spacings between the stripes follow a De Bruijn sequence (Figure 6a,b). A color camera is used to visualize the deformed grid and the correspondence problem is solved by a geometric constraint (epipolar constraint). Only a qualitative assessment of the result can be done, and low spatial resolution is gained, missing details (Figure 6c). 

**Figure 7:**
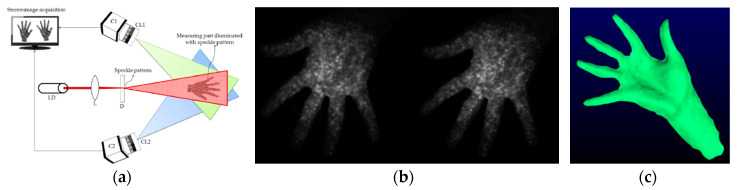
3D hand reconstruction carried out in [63], (**a**) system setup, (**b**) captured stereo images, (**c**) reconstructed 3D model of the hand.

In [63], a one-shot technique is proposed for the 3D reconstruction of human body parts (Figure 7a). A laser speckle pattern is projected and a single pair of images, captured by two synchronized cameras, is used (Figure 7b). The proposed system employs KLT (Kanade Lucas Tomasi) feature tracking method to locate the corresponding points differently to conventional laser speckle 3D measurement systems that realize stereo correspondence by digital image correlation (DIC). Results evidenced 3D point clouds containing no outliers and the authors claimed the achievement of a sufficient quality of the 3D reconstruction (Figure 7c). The proposed technique achieved an accuracy of 0.393 mm in measuring human hands, significantly better than the measurement accuracy (0.845 mm) obtained by using a Kinect v1. 

However, all these studies are focused on the development of real-time structured light techniques, which have their merits and shortcomings, and the acquisition of human hands is used to prove the effectiveness of the implemented approaches, without carrying out any dimensional assessment. Thus, it was not possible to quantitatively determine the achieved accuracy.

All the described techniques are based on a scanning unit, which is composed of a light source (i.e., a laser or a digital projector) and one or two cameras. The unit is typically mounted on a tripod and it is moved around the human body part, while the patient remains still, to obtain different viewpoints. The type of movement may vary according to the body part that must be acquired. The overall acquisition time must consider not only the time needed for the single scan but also the time spent to manually place the scanner around the subject. Moreover, the single scans, obtained by different viewpoints, must be aligned in a common reference system to gather the overall patient’s anatomy. Stationary scanners are characterized by good performances in terms of accuracy and reliability [2]. However, their use is limited by the high costs and the need for skilled technicians to acquire anatomical data.

**Figure 8:**
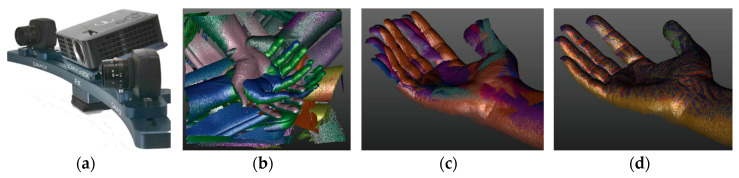
3D hand reconstruction carried out in [73], (**a**) structured light scanner, (**b**) point clouds from eight different viewpoints, (**c**) aligned point clouds, and (**d**) deformable registration of the point clouds to compensate motions.

Scientific literature also documents the use of commercial stationary scanners in clinical practice for the acquisition of the 3D hand/wrist shape. For example, the structured-light optical 3D scanner Scan-in-a-Box (OpenTechnologies Srl, Brescia, Italy) has been used in [73] to acquire the lower part of the limb (Figure 8a). This lightweight and low-cost reconfigurable scanner, composed of a multimedia projector and two digital cameras, performs high-resolution structured-light scans in about 4 s, guaranteeing a metric accuracy till 0.1% with respect to the object size (for the hand reconstruction reported in [73], about 0.2 mm). The study aimed to discuss the key aspects to produce highly customized hand orthoses. The scanning process required about 2 min to reposition the scanner around the limb to collect eight different point clouds from different viewpoints (Figure 8b). The various point clouds required an alignment into a common reference frame (Figure 8c) and a deformable registration [74] was carried out to compensate for motions (in particular of the fingers) that occurred during the acquisition stage (Figure 8d).

The REXCAN4 (Solutionix, Korea) has been used in [75] to outline a novel methodology for the design and manufacturing of a customized wrist orthosis. The REXCAN4 is an industrial structured light scanner composed of two cameras and a projector and it is based on a phase-shifting approach. The system performs a single scan in about 1 s. A table summarizing the main features of the cited equipment, and other similar commercial devices, is reported in Appendix A. 

### 3.2. Hand-Held Real-Time Scanners

Hand-held (portable) scanners are typically real-time devices, which operate as video cameras. When real-time scanning is performed, the most challenging aspect is the alignment between consecutive scans. This process is generally carried out on a frame-by-frame approach exploiting iterative closest point (ICP) algorithms which can be enhanced by implementing IMU sensors. Hand-held scanners were first developed to scan objects and scenes, but they have all the potentialities to be used also in the scanning of human body parts. The use of portable optical scanners seems to represent a suitable solution for the scanning of upper limb anatomy in terms of digitization speed, cost, accuracy, and ease of use. Moving the hand-held scanner around the target surface, to collect 3D data from different views, is easier than moving a stationary scanner mounted on a tripod.

Two different typologies of portable scanners can be considered: high-end and low-end setups. They can be used for the same applications in 3D scanning even if they differ for accuracy and cost. In particular, the cost of high-end portable scanners represents a limit for their use in small clinical centers. Among low-end portable devices, the advent of affordable consumer-grade depth cameras (RGB-D cameras) has brought a significant advancement of visual scene reconstruction methods [13] and their use for 3D reconstruction of human body parts has become increasingly consolidated within biomedical applications [76]. 

#### 3.2.1. High-End Portable Scanners

**Figure 9:**
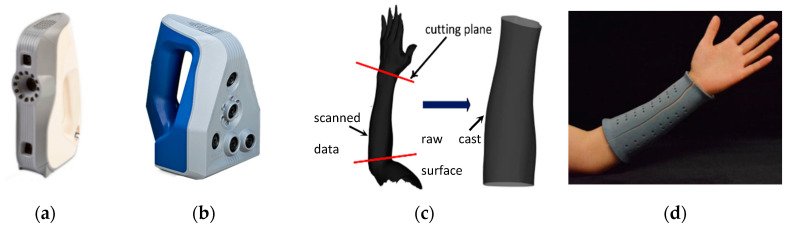
Upper limb reconstruction carried out in [77], (**a**) Artec Eva (Artec 3D, Luxembourg) hand-held scanner, (**b**) Artec Space Spider (Artec 3D, Luxembourg) hand-held scanner, (**c**) limb scanned data and raw cast surface cut from the data, (**d**) final 3D printed cast.

**Figure 10:**
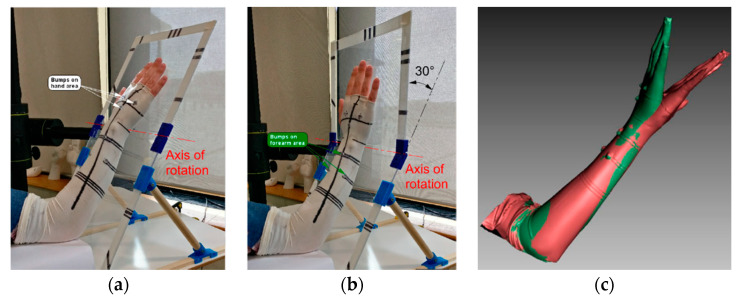
Upper limb reconstruction carried out in [78], (**a**) scanning setup with the upper limb in neutral hand posture; (**b**) scanning setup with the upper limb in extended hand posture, (**c**) 3D scans in neutral and extended hand postures.

Scientific literature reports an extensive use of high-end portable scanners, which are characterized by high accuracies, great versatility, and ease of use. In [77], the Artec Eva (Figure 9a) and the Artec Space Spider (Artec 3D, Luxembourg) (Figure 9b) scanners have been used to scan an injured limb to develop an effective modeling process to create patient-specific and hygienic orthopedic casts by 3D printing technologies (Figure 9c,d). These two portable scanners are high-resolution scanners. The Artec Eva scanner is based on a structured light technology and is characterized by a working distance of 400–1000 mm, a data acquisition speed of about 2 million points/s, a resolution up to 0.5 mm, and 3D accuracy up to 0.1 mm. The Artec Space Spider scanner is based on blue structured light technology and is characterized by a working distance of 200–300 mm, a data acquisition speed of about 1 million points/s, a resolution up to 0.2 mm, and a 3D accuracy up to 0.05 mm. The Artec Eva scanner has been also used in [78] to develop and assess an upper-limb 3D scan hand posture-correction procedure. The scanning speed was set at 15 frames per second to guarantee a proper overlap between consecutive scans. A bespoke jig was designed to support the upper limb in two controlled hand postures (neutral and extended) during the 3D scanning, without repositioning the limb (Figure 10a,b). The 3D scanning procedure showed that the construction of the jig allowed it to efficiently capture the upper-limb anatomy from dorsal and palmar sides and generate a whole upper-limb 3D scan geometry (Figure 9c). The same device was also used in [79] to scan the upper limb anatomy of a patient with congenital absence of the thumb to design a customized thumb prosthesis.

#### 3.2.2. Low-End Portable Scanners

The interest of the medical community in low-cost commercial devices is related to the necessity of obtaining the 3D patient anatomy easily and quickly even in small satellite clinical centers rather than only in highly specialized institutes. The recent introduction of consumer-accessible depth cameras (range cameras or RGB-D cameras) has opened a wide variety of interesting possibilities for medical applications. RGB-D cameras are characterized by a limited cost and a high frame rate.

Range sensors (also known as RGB-D cameras) are usually composed of an RGB (red-green-blue) camera, an infrared (IR) camera, and an IR projector. These devices can acquire both surface geometry and color texture of the human body by imaging the patterns projected on the body surface. The projection can be composed of a single pattern or a temporal sequence of fringe patterns. The patterns are used for the scene coding and the reconstruction of surface geometry by triangulation. It is worth noting that if multiple devices are simultaneously used, the interference between the patterns projected by the different light sources could impair the accuracy of the scanning results.

A low-cost short-range depth camera, specifically designed for hand applications, is the Carmine 1.09 (Primesense, Israel), which has been used in many studies for hand pose estimation [80,81]. This consumer RGB-D camera uses structured light by projecting a speckle pattern over the scene. However, the most popular of these devices, introduced by Microsoft in 2010, is the Kinect V1 (based on the triangulation principle) and Kinect V2 (based on the time of flight principle), which have been commercialized since 2010. The Microsoft Kinect V1, based on the Primesensor™ design by Primesense, exploits a structured light technology and uses a color camera, a monochrome NIR (Near InfraRed) camera and a NIR projector to project a speckle pattern. Depth estimation of the scene is carried out at 30 fps. The baseline between the projector and the NIR camera is about 75 mm. The Microsoft Kinect V2 is instead based on the TOF principle and exploits the modulation of an IR light source with a square wave and a flash camera. The time needed by the light for reaching each camera pixel of the flash camera is determined by detecting the phase shift between the square wave and the signal received by each camera pixel.

The two Kinects were primarily developed to be fixed in the environment to capture full-body motion. However, their use was soon opened also to the inverse task, which is the reconstruction of a static environment by moving the sensor and collecting surface information as 3D data. In this sense, KinectFusion was the first project-oriented to the real-time fusion of all the depth data streamed from a moving Kinect sensor into a single implicit surface 3D model [82,83]. A metrological comparison between Kinect I and Kinect II sensors has been carried out in [23] by using standard objects based on spherical and cubic geometries. Results showed precision values between 2 and 6 mm for both devices at a working distance of 1 m. On the other hand, for working distances of 2 m the precision of Kinect V1 increased up to 12 mm while Kinect V2 kept all values below 8 mm. Additionally, accuracy values attested better results for Kinect V2 (<5 mm) for working distances between 1 and 2 m while Kinect V1 showed accuracy values of 12 mm at working distances of 1 m and 25 mm at working distances of 2 m.

**Figure 11:**
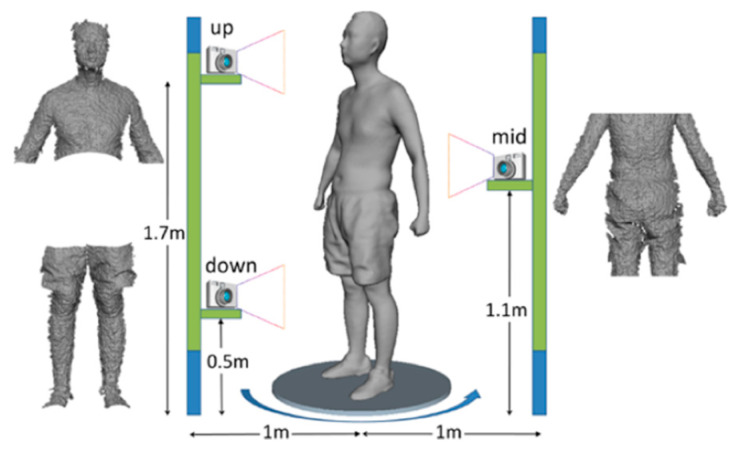
System setup, composed of three Kinect sensors, proposed in [85] to capture human body parts without interference problems.

Moreover, several studies have investigated the possibility of using this device as a 3D acquisition system for human body scanning. In [84], a Kinect V1 sensor was used to obtain a virtual model of the upper limb anatomy for the assessment of upper limbs lymphedema through volume measurements. In [85], a scanning setup composed of multiple Kinects (V1) has been developed to capture 3D full human body anatomies. Two Kinects were used to reconstruct upper and lower parts of the body without overlapping regions, while a third Kinect was used to capture the middle part of the human body from the opposite direction, to avoid interference phenomena (Figure 11). The patient is then placed on a rotary plate, to achieve a 360° reconstruction. The three range cameras are synchronized and mutually calibrated so that the captured data are aligned into a common reference frame. 

A further low-cost 3D scanner is represented by the Structure Sensor (Occipital, Inc., Boulder, CO, USA), which was developed for the 3D reconstruction of environments by a simple connection to an iPhone or an iPad (Apple, Inc., Cupertino, CA, USA) with the aim of having a user-friendly and hand-held device. The sensor is based on the projection of a speckle near-IR pattern and exploits the triangulation principle. A newer version of the structure sensor has been recently released with the name of Mark II. This new version is equipped with an inertial measurement unit (IMU), which makes tracking more robust, and is characterized by a higher resolution depth (1280 × 960 vs. 640 × 480 of the structure sensor). 

In [86], the Structure Sensor, integrated with an iPad (Apple, Cupertino, CA, USA), has been used to quantify the static posture in a clinical environment to evaluate the effectiveness of physiotherapy treatments, spinal orthoses, or surgical treatments. The study concluded that the approach turned out to be a relatively inexpensive method to quantify reliable postures and postural deformities, thus representing a feasible alternative to motion analysis systems, which are expensive and require a high degree of technical experience. In [87], the Structure Sensor has been used to define a low-cost and hand-held 3D scanning process for the assessment of breast surfaces in view of plastic and reconstructive surgery. The study reported analogous practicability and reliability for surface acquisition with respect to clinically established scanners as the Vectra M5 Scanner (Canfield Scientific Inc., Parsippany, NJ, USA), which is a stationary passive stereo photogrammetry-based system, and the Artec Eva 3D scanner (Artec3D, Luxembourg, Luxembourg). In [88], the Structure Sensor has been used to reconstruct 3D hand models in functional hand postures with the aim at creating 3D hand parametric models to be used in the design of customized short thumb orthoses.

**Figure 12:**
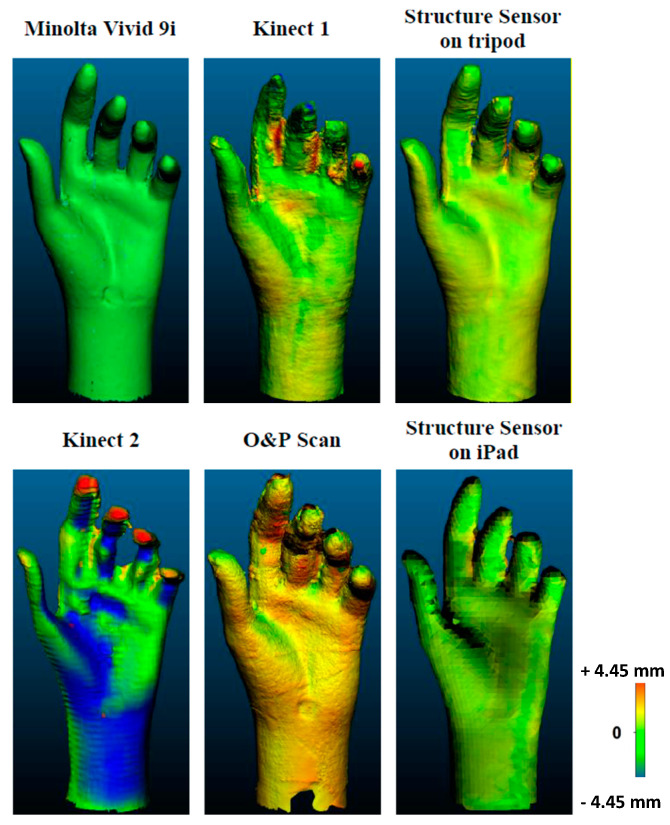
Results obtained by the different devices compared in [89]. The model acquired with the Minolta system has been used as a reference for the comparison of all the other devices.

In [89], a comparison between four low-cost 3D scanners (Kinect V1 and V2, the Structure Sensor and the O&P Scan Rodin4D) and a high-end laser scanner, Minolta Vivid 9i (Konica Minolta), was carried out for the reconstruction of human body parts. Three anatomical parts of a mannequin (a hand, a thigh, and a chest) were used to investigate the results on different dimensions and detail levels. The study concluded that the Minolta Vivid 9i has the best resolution. On the other hand, the type of technology makes it inadequate for human body scanning, since it requires multiple scans taking minutes to be completed and it is not easy to move around the patient’s body. Thus, the patient would move also varying the posture of the parts to be acquired, due to both joints and soft tissue movements. The best low-cost device resulted to be the Structure Sensor (as shown in Figure 12) for the low deviation of the results with respect to the reference models and also for the possibility to move around the objects without any wire and the need of an additional tracking system.

Intel^®^ (Intel Corporation, Santa Clara, CA, USA) has released the “RealSense™” family, which is a competitive triangulation-based range sensing technology [90], implemented in standalone consumer-grade units as the RealSense SR305, the RealSense D415, and the RealSense D435. The SR305 model, based on the SR300 depth module, implements a short-range (SR) coded light approach by projecting a sequence of coded light patterns. The unit is equipped with an infrared laser projector, an infrared camera, and a 2M pixel RGB camera. The effective depth range is optimized in the range of 200–1500 mm for indoor use. The dimensions of the device are approximately 139 × 26.13 × 12 mm and the weight is 9.4 g. In [91], a systematic evaluation of the metrological performances of the SR300 has been carried out by applying the VDI/VDE normative. The study concluded that although the device was mainly developed for applications such as tracking, gaming, or gesture recognition, it could be also used as a low-cost device for 3D scanning applications dealing with health, fashion, and cultural heritage. The RealSense D415 and D435 depth cameras are based on an active stereo vision approach, exploiting two IR cameras (baseline of 55 mm). An infrared projector is used to project a static pattern, which enhance texture of low-texture scenes. A color camera, with a resolution of 1920 × 1080 pixels, is also added to provide color texture information to be superposed on the depth data. The two devices mainly differ in the field of view angles and in the exposition time of the camera-integrated shutter. The Intel RealSense D415 depth camera has a narrower field of view (FOV, approximately 65°) with respect to the Intel RealSense D435 depth camera (approximately 85°). For this reason, the Intel RealSense D415 has a higher pixel density, thus resulting in a higher resolution, while a greater FOV allows minimizing blind spots, thus facilitating robotics applications. Thereby, when accuracy is paramount, the Intel RealSense D415 promises to provide better results, especially when used at a short-range (i.e., <1 m). Metrological characterization of the Intel RealSense D415 stereo depth camera has been carried out in [92]. The study demonstrated that the D415 model is fully comparable to the SR300 model in terms of errors assessed through the VDI/VDE standard. The device is also in line with the results obtained with other comparable devices in the scientific literature and exceeds their performance if the built-in filtering algorithms are exploited. The main characteristics of hand-held portable scanners are reported in Appendix A.

#### 3.2.3. Comparison between Hand-Held and Stationary Scanners

Scientific literature reports many studies focused on the comparison between different scanning architectures. In particular, works are mostly dedicated to the comparison between handheld and stationary scanners, which are often used as a reference. In [93], the performances of three hand-held portable scanners (zSnapper, Artec MH, ZScanner 700) and two stationary tripod scanners (ATOS I, ATOS TripleScan, both manufactured by GOM, Braunschweig, Germany) have been compared to find a suitable 3D scanner for the digitization of human body parts in the prosthetics and orthotics fields. The performed measurements highlighted that in the case of plaster cast digitization, high-resolution stationary scanners give more reliable results. The direct patient scanning, instead, takes advantage from the use of handheld scanners since they are less affected by the patient’s movements. In particular, the study concluded that Artec scanner can be a good trade-off in terms of ease of use, measurement speed, data processing, and quality of the 3D data.

**Figure 13:**
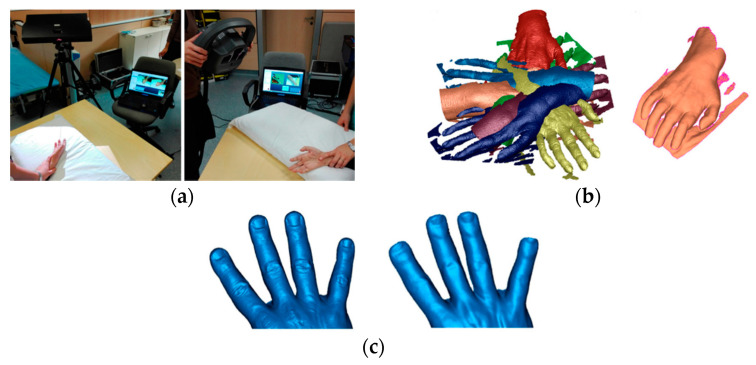
Upper limb reconstruction carried out in [94], (**a**) scanning setup with the Cronos 3D Dual (left) and Insight 3 (right), (**b**) raw scanned data stationary scanner (left) and real-time scanner (right), (**c**) comparison between stationary (left) and hand-held scanner (right) in terms of surface details.

Further research recently focused on the 3D scanning of hand anatomy, including fingers, for the design of customized orthoses [94]. This work investigated the performances of two distinct typologies of structured light scanners: Cronos 3D Dual and Insight3. Both the scanners are manufactured by Open Technologies Srl, (Brescia, Italy). The first one is a high-accuracy stationary scanner, based on the projection of a sequence of structured light patterns, capable of acquiring a single scan in 4 s, while the second one is a hand-held scanner capable of performing real-time acquisitions by projecting a fixed structured light pattern. The two different scanning procedures (Figure 13a) were compared in terms of acquisition time, completeness of anatomical data, presence of motion artefacts, and time required for post-processing. Both scanners proved to be appropriate for hand anatomy acquisition for orthotic applications. However, motion artefacts, due to involuntary movements of the patients, affected the acquisition by the stationary scanner, which had to be corrected by a deformable alignment technique [74]. On the other hand, motion artefacts were reduced by using the real-time scanner due to the faster acquisition time (Figure 13b). The accuracy in the detection of fine details (folds, veins, knuckles, wrinkles) is higher for the stationary scanner with respect to the real-time scanner (Figure 13c). However, the difference in terms of surface detail achieved by the two scanning technologies is not expected to be a discriminating factor in the creation of customized orthoses using CAD and/or mesh editing and AM technologies: this process indeed requires good metric accuracy but not at the level of high-fidelity skin texture reproduction. 

In [95], an overview of the application of different 3D scanners for Prosthetic and Orthotic Clinical Practice is given. Two low-end portable scanners, three high-end portable scanners, the stationary scanner ATOS Core 135 (GOM), and the body scanner DI3D FCS-100 (Dimensional Imaging) have been used. The low-end portable scanners are the Sense 3D scanner (3D Systems, Rock Hill, SC, USA) and the Structure Sensor (Occipital, Inc., Boulder, CO, USA). The high-end portable scanners are the Artec Eva scanner (Artec 3D, Luxembourg, Luxembourg), the Go!SCAN 3D scanner (Creaform Inc., Lévis, QC, Canada), and zSnapper Portable scanner (VIALUX, Chemnitz, Germany). The study concluded that clinical practice greatly benefits from non-contact 3D anatomy reconstructions, but there is no single scanner that can fulfill all the requirements. 

### 3.3. Photogrammetric Body Scanners for the Upper Limb Scanning

Body scanners refer to 3D scanning systems that use multiple sensors to capture 3D geometry from different angles at the same time or in a short sequence. Subjects must remain still during the scanning process. They are usually designed to create an enclosed space, like a dressing room, to ensure the subject’s privacy. Body scanners are mainly based on photogrammetry.

**Figure 14:**
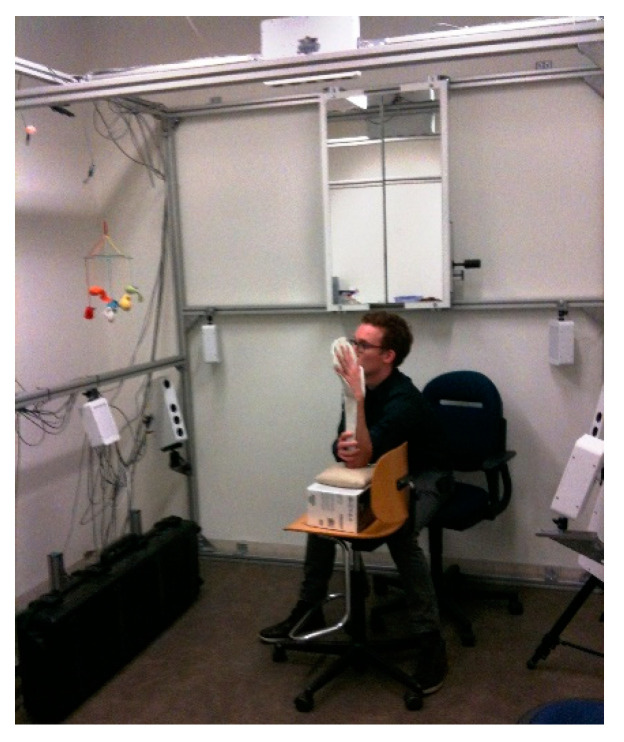
3dMDface system used in [97] for hand acquisition.

Many commercial solutions exist on the market and are widely used for healthcare applications. The 3dMDhand System (3dMD LLC, Atlanta, GA, USA), for example, was used in [96] to generate accurate 3D surface meshes for any subject’s hand in a wide array of motions and poses. This system is composed of five scanning units, each containing one color camera with resolution 2448 × 2048, two grayscale cameras with resolution 1624 × 1236, and two speckle projectors that provide the illumination for the grayscale images. An industrial-grade flash system assures the synchronization of the capturing process. A 3dMD system (the 3dMDface) has been also used in [97] for the acquisition of 30 soft tissue landmarks of the hand. The photographs were taken with each subject seated with flexion in the elbow joint and the hand kept in a predesigned template with the fingers in a fully abducted and extended position (Figure 14). The template was removed by an assistant, less than 1 s before the photograph was taken, to achieve accurate images of both the palmar and dorsal aspect of the hand. The patient was instructed to keep the hand still and not to move the hand to another position after the removal of the template. The study concluded that accurate and reproducible 3D models of the hand could be obtained and confirmed the potentialities of 3D photogrammetry in the soft tissue analysis of the hand for pre- and postoperative evaluation of reconstructive trauma surgery, aesthetic surgery, and for educational purposes. 

**Figure 15:**
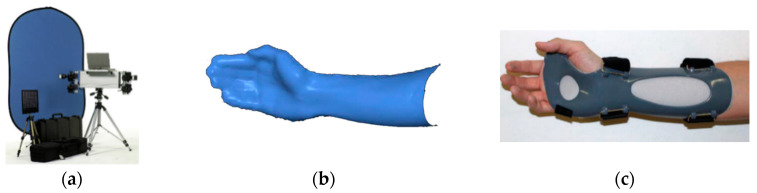
Upper limb reconstruction carried out in [98], (**a**) Di3D FCS-100 system; (**b**) 3D model of the limb after smoothing, (**c**) final orthosis.

In [98], the Di3D FCS-100 system (Dimensional Imaging Ltd., Glasgow, Scotland, UK) has been used to acquire the one-side geometry of the upper limb to design a customized wrist orthosis. The Di3D FCS-100 is a passive stereophotogrammetric system specifically designed to capture the 3D shape of body parts (Figure 15a). It uses four 10 megapixels cameras to create 3D models with highly detailed texture maps. The photographs are taken at the same moment and the final 3D model is created in a few seconds. Only a single scan from one side was required since a one-side type orthosis was needed (Figure 15b,c). The whole limb scan by the Di3D FCS-100 system would have required the acquisition and alignment of multiple scans from different points of view, making the overall process more complex.

**Figure 16:**
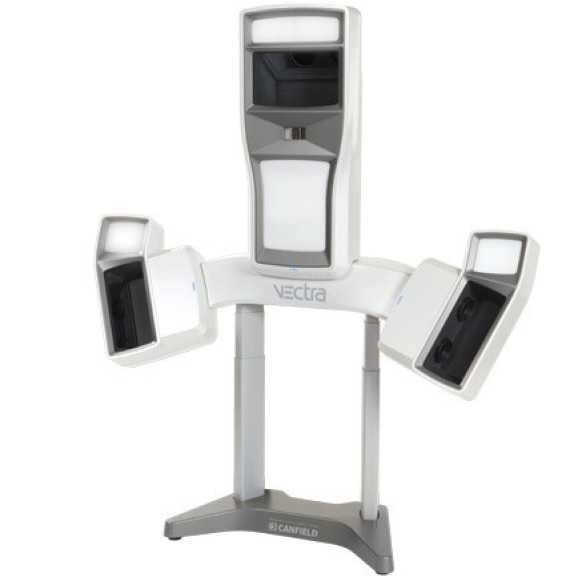
The Vectra 3D imaging system used for the upper limb reconstruction.

In [99], the Vectra 3D (Canfield Scientific Inc., Parsippany, NJ, USA) imaging system (Figure 16) has been used to obtain volume measurements of the upper limb in patients with lymphedema. Vectra 3D system is a stationary passive stereophotogrammetry-based system. The system stands on the floor and it is equipped with a motorized lift to adjust for patient height. The study concluded that the Vectra 3D system provides reliable data to estimate limb volume compared to the most adopted techniques (tape measurements and water volume measurements).

**Figure 17:**
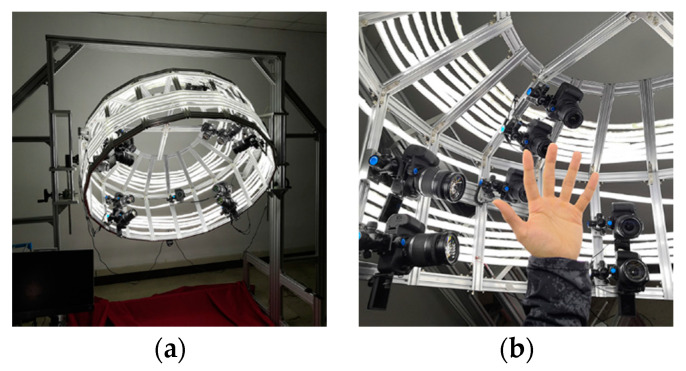
Photogrammetric systems developed in [100] for the creation of 3D hand models, (**a**) system setup, (**b**) hand pose during the acquisition process.

In [100], a photogrammetry approach is proposed to capture both hand shape and pose to create a public dataset composed of high-quality hand color images, 2D hand landmarks, and 3D point clouds. The multiview system is composed of 15 synchronized digital single-lens reflex (DSLR) cameras, which can acquire high-quality 4K resolution color images. The cameras are arranged within a circular cage and LED stripes are mounted outside the cage to provide a shadow-less illumination around the hand (Figure 17). Nonrigid iterative closest points algorithm is performed to drive a template hand to fit the point clouds and register all the hand meshes singularly acquired. The main characteristics of body scanners are reported in Appendix A. 

### 3.4. Custom SL Scanners for Upper Limb Reconstruction

Most of the devices described in the previous sections have been originally developed for industrial fields (except for the body scanners) and only later adapted to medical applications. Only a few scanners have been specifically developed for hand scanning. The main idea at the basis of these customized hand scanners is to employ multiple devices to simultaneously acquire the anatomy from different viewpoints, thus minimizing involuntary movements of the target surface but with a smaller footprint compared to whole-body scanners.

**Figure 18:**
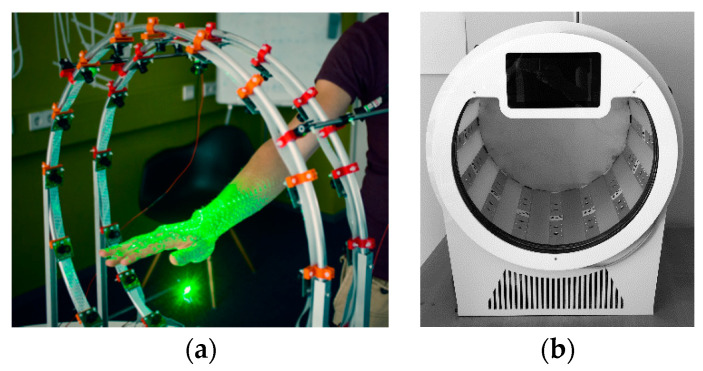
3D hand scanner “Curatio”: (**a**) concept prototype, (**b**) consumer-grade final 3D hand scanner.

Researchers at the Delft University of Technology developed a 3D hand scanner able to scan a human hand [101] (Figure 18a). The system, which is commercialized with the name “Curatio” (Vectory3, The Hague, The Netherlands) [102] (Figure 18b), is based on laser pointers and multiple cameras, which can acquire pictures at the same time, arranged in a cylindrical frame. This system enables an instant reconstruction of the patient’s hand.

**Figure 19:**
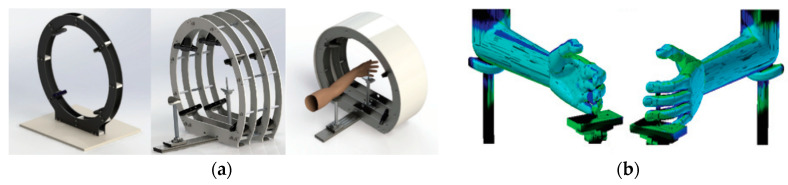
3D upper limb scanner developed in [103,104]: (**a**) concept prototype, (**b**) results of the aligned four different scans.

A low-cost 3D scanner has been developed in [103] by assembling four Intel RealSense SR300 depth cameras to study the feasibility of acquiring human hand–wrist–arm anatomy. The depth cameras are arranged on a circular rig (Figure 19a) to acquire the whole geometry in a single shot (Figure 19b). Although the RealSense SR300 allows fast single acquisitions (60 fps), the use of multiple devices at the same time leads to interference problems since the SR300 module is based on the projection of a temporal sequence of fringe patterns on the scene. If one or more cameras partly share the same field of view, the patterns projected by different cameras interfere with each other, thus degrading the acquired data. For this reason, the overall acquisition must be carried out by sequentially turning on the projector laser intensity for every single camera. This process leads to an overall acquisition time of 1.2 s. This preliminary study, however, gave promising results in the definition of a fast and fully automatic procedure for the surface reconstruction of human hand anatomy. In [104], a greater field of view was obtained by adding a second circular rig, with other four Intel RealSense SR300 depth cameras, placed adjacent to the first one. The eight-cameras device was used to reconstruct hand–wrist–arm anatomy for the computer-aided design of custom 3D printable casts for wrist fractures.

**Figure 20:**
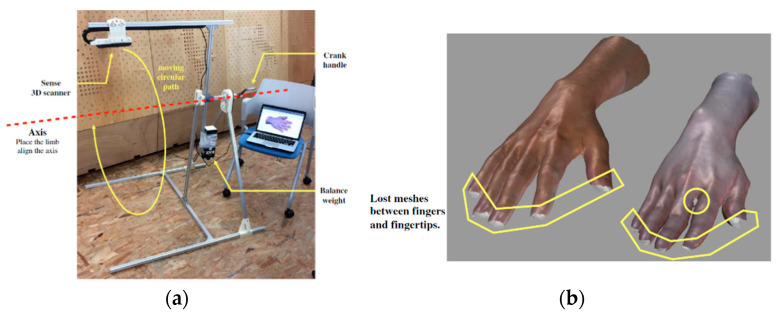
3D upper limb scanner developed in [105]: (**a**) system setup, (**b**) obtained results highlighting 3D data loss in correspondence of fingertips and between fingers.

A similar attempt has been made in [105] by developing a scanner mount that makes possible the digitization of the upper limb from different viewpoints. The aim of this study relies on the development of a parametric model as the design generator for 3D printed orthoses. For this purpose, the Sense 3D scanner (3DSystems) was placed on a rotating arm connected to a mount made by aluminum rail sticks. The scanner could then be rotated along a circular pattern around the patient’s limb, maintaining a fixed working distance from the target surface, by smoothly rotating the crank handle (Figure 20a). The overall scanning process takes about 20 s, even if a few details between the fingers and in correspondence of the fingertips could not be acquired (Figure 20b). 

### 3.5. 3D Scanning Architectures Comparison

The described scanning technologies are implemented in several architectures. The comparison of the main architecture families is reported in terms of handling, acquisition time, accuracy level, and cost (Table 2). Data are reported as ranges since the exact values strongly depend on the specific devices. As can be noted, most of the devices exploit SL approaches, which provide the best combination of accuracy, robustness, and resolution (as evidenced in Table 1), especially in the case of multiple frames methodologies. Nevertheless, since these methods suffer from a long acquisition time, many solutions in the medical field rely on single frame SL, time of flight or photogrammetry methods, accepting a compromise between acquisition accuracy and speed. To this extent, it is worth noting that body scanners are based on multicamera photogrammetry approaches, thus allowing a short acquisition time, which is only limited by the camera shutter time. Nevertheless, this is not directly related to the frame rate, meaning that even if the single-frame acquisition requires few milliseconds, the acquisition of multiple frames could be much slower, not allowing gestures scanning. Additionally, the processing time is high, thus the acquisition results cannot be evaluated in real-time. 

Table 2 also highlights that a large family of handheld scanners is available, weighing less than 1 kg, thus allowing for the development of multisensor architectures to enhance the overall performance. These devices provide fast scanning capabilities at low cost, at the expense of lower accuracies.

## 4. Discussion

### 4.1. State of the Art of Upper Limb 3D Scanning

Optical scanning represents a suitable technology for all those applications requiring high resolution and accuracy [11]. Moreover, parameters such as low invasiveness, speed, and portability are of utmost importance when a patient’s anatomy must be reconstructed. Human body scanning, and particularly upper limb (i.e., hands, fingers, wrists, and forearms) scanning, is a complex task that requires solving problematic aspects. The 3D shape of the hand leads to self-occlusions, for this reason, multiple scans from different points of view are required. Moreover, it is difficult to keep the hand in a stable position and the results are usually affected by artefacts due to involuntary movements. Many approaches have been proposed, as documented in the previous sections, but critical issues remain. In most situations, a subject cannot maintain the hand static for the whole duration of the scanning process. For this reason, scanning speed is a relevant key factor, even at the expense of a potential loss of accuracy and resolution, since nonvoluntary patient movements can lead to artifacts in the scanning results. These movements can be divided into two different typologies [94]: (1) intrascan movements, which are those occurring during a single scanner acquisition and (2) interscan movements, which are those occurring between different scanner acquisitions consecutively carried out. The first movement typology affects the results of any scanner type and can only be minimized by speeding up the single acquisition time (e.g., adopting single-shot approaches). The incidence of the second movement typology, instead, is directly related to the number of scans required to reconstruct the entire anatomy and the time spent to reposition the scanner around the target surface. Thus, a real-time single-shot 3D scanning technique would be preferable for most situations [17]. Therefore, the popular structured light method based on the phase-shifting technique cannot be considered properly suitable for human body acquisition, since it needs to project at least three images. Moreover, the use of stationary scanners is not fully advisable since they cannot be easily handled (because of their weight) and their movement around the patient arm slows down the acquisition process and requires a proper operational space. Portable solutions, like those represented by hand-held scanners, have the advantage of being more lightweight with respect to stationary scanners and can be easily managed by the operator. Among this scanner typology, many high-end solutions exist, which can provide accurate real-time scanning guaranteeing the alignment between consecutive scans on a frame-by-frame basis through an ICP algorithm. The main drawback of this solution relies on the cost of such devices, which is still too high and limits their use in highly specialized clinical centers. In recent years, the introduction of RGB-D cameras has allowed the definition of scanning procedures of human body parts by low-cost equipment, opening a wide variety of interesting possibilities for medical applications. The advantages of using low-end portable scanners (i.e., Structure sensor, Kinect, Intel RealSense) certainly rely on the portability, easy handling, and the limited cost. Among the plethora of commercial devices, the Intel RealSense depth cameras proved to be one of the most suitable devices, providing a good trade-off between cost, ease of use, compactness, and accuracy. Moreover, the availability of Software Development Kit resources allows for the customization of the acquisition software. Although the use of portable scanners certainly speeds up the acquisition process with respect to stationary scanners, small movements of the upper limb anatomy may still affect the scan results.

An alternative could be represented by body scanners, which can reconstruct whole body shapes in a very short time. However, when body scanners are used, a key problem is represented by the hand size, which is small in comparison to the full-body, and thus the acquisition often results in an insufficient resolution. Moreover, body scanners are based on architectures characterized by a low level of portability and flexibility and high costs. For this reason, some multiview-based custom solutions have been proposed to minimize the acquisition time by using multiple devices at the same time. This multiview single-shot data collection approach is less affected by involuntary movements of the targets if compared to portable scanners. However, a limitation is given by the fixed number of preconfigured viewpoints, which, in some cases, could cause some difficulties in the acquisition of complex geometries due to occlusion problems.

### 4.2. Trends and Future Challenges

The presented review highlights that all the available technologies are not free from drawbacks, thus allowing multiple research activities to address challenging applications. 3D optical scanning is continuously evolving, as exemplified by the recent market release of new sensors, such as Azure Kinect and RealSense LiDAR camera. Even if these specific devices have not been yet tested in the acquisition of human upper limb, they seem promising, and some experimental studies would be of great interest. Additionally, it is worth noting that many lightweight sensors provide fast acquisitions at low cost: this encourages the development of architectures, which combine multiple identical sensors (as done in [103,104]) but in a handheld fashion, thus enlarging the scanner field of view and working volume, shortening the acquisition time and allowing for fast 360° reconstruction of complex shapes.

Several other sensor architectures could be of interest to the scientific community, integrating sensors based on different technologies to enhance the acquisition outcomes, e.g., improving and automatizing the data registration process. To this extent, IMU sensors could be coupled with handheld scanners, to provide a first guess for the data registration during continuous scanning. Additionally, some specific sensors for the hand gesture tracking are available at a low cost, such as the Leap Motion Controller [106]. Even if these sensors do not perform a 3D scan of the arm surface, they can automatically detect some specific features, such as knuckles, which could be used to assess the rotation between different scanning orientations, thus providing a rough alignment between data captured from different viewpoints. Finally, RGB-D cameras also provide 2D images together with 3D scan data. These images could be processed by photogrammetry algorithms, to obtain rough 3D models of the target anatomy that could be used as a reference to align scan data, which are more accurate and less noisy than photogrammetric reconstructions.

## 5. Conclusions

This paper presentdd a review of technologies and sensor architectures for the 3D scanning of upper limb anatomies. The main working principles at the basis of existent devices were described, proposing also a categorization based on handling, pre/post-processing effort, and potentialities in real-time scanning. A further distinction was also made between research approaches and commercial sensors: the former representing prototypes developed for specific applications, the latter representing the most popular off-the-shelf solutions for 3D scanning, grouped into different cost ranges. The different solutions were also compared in terms of scanning performances and applicability to different scenarios. In particular, the most effective sensors for human body scanning were found to be hand-held low-cost devices, which allow for fast single frame acquisition and provide a software development kit for acquisition and post-processing customization.

Most of the reviewed papers were concerned about evaluating the performances of the single-frame acquisition process, without providing rigorous insights about pre/post-processing (e.g., sensor calibration, point cloud registration), which, instead are crucial issues in determining the final result. Finally, in the authors’ opinion, the integration of different sensors and technologies represents the most promising approach to overcome limitations of every single device, thus representing an interesting research activity for the development of customized 3D scanning architectures.

## Figures and Tables

**Table 1:** Comparison between 3D scanning technologies.

Technology	Method	Robustness	Resolution	Processing	Real-Time	Accuracy
Time-of-Flight	TOF	Medium	Low	Simple	x	Low
	Lidar	High	Medium	Simple	x	Low
Passive Image-Based	Depth from shading/focus	Low	Low	Complex	-	Low
	Stereo vision	Low	High	Medium	x	Medium
	Photogrammetry	Low	Low	Complex	x	Low
Structured Light-Based	Multiple frames	High	High	Simple	-	High
	Single frame	Medium	Medium	Medium	x	High

**Table 2:** Comparison between 3D scanning architectures.

Architecture	Technology	Handling	Acquisition Time	Accuracy	Cost
Stationary scanners	SL	Low 3–15 kg	Long ≈5 s	Very high 0.02–0.1 mm	Very high 20–80 k€
High-end portable scanners	SL	Medium <3 kg	Short ≈1 s	High 0.05–0.1 mm	High 14–30 k€
Low-end portable scanners	SL, TOF	High <1 kg	Very short 1/30–1/60 s	Low ≈1 mm	Low 100–500 €
Depth cameras	SL	Very high <0.1 kg	Very short 1/60–1/90 s	Low ≈1 mm	Low 80–150 €
Body scanners	PG	Very low >15 kg	Very short 1.5–3.5 ms	Low 0.2–1.5 mm	High 15–50 k€

## Appendix A

**Table A1:** Stationary scanners.

Device		
**Scan-in-a-box**	Technology	Structured light
	Camera resolution (pxl)	2M
	Point scanning (mm)	0.08/0.4
	Max accuracy (mm)	0.08
	Working distance (mm)	200–1100
	Scanning area (near)/(far)	100 × 80/500 × 400
	Scanning time (s)	4
	Weight (kg)	3
	Dimensions (mm)	400 × 105 × 92
	Cost (k€)	3–5
**Cronos 3D Dual**	Technology	Structured light
	Camera resolution (pxl)	1600 × 1200
	Point scanning (mm)	0.09/0.18
	Max accuracy (mm)	0.02
	Working distance (mm)	400–1000
	Scanning area (near)/(far)	150 × 115/300 × 225
	Scanning time (s)	4
	Weight (kg)	7.2
	Dimensions (mm)	540 × 250 × 145
	Cost (k€)	20–40
**Minolta Vivid9i**	Technology	Laser triangulation light block method
	Camera resolution (pxl)	640 × 480
	Point scanning (mm)	0.145–2.3
	Max accuracy (mm)	0.1
	Working distance (mm)	500–2500
	Scanning area (near)/(far)	93 × 69/1495 × 1121
	Scanning time (s)	2.5
	Weight (kg)	15
	Dimensions (mm)	221 × 412 × 282
	Cost (k€)	25–55
**Minolta Range7**	Technology	Laser triangulation light block method
	Camera resolution (pxl)	1280 × 1024
	Point scanning (mm)	0.08–0.28
	Max accuracy (mm)	0.08
	Working distance (mm)	450–800
	Scanning area (near)/(far)	99 × 79/334 × 267
	Scanning time (s)	2
	Weight (kg)	6.7
	Dimensions (mm)	295 × 190 × 200
	Cost (k€)	80
**REXCAN4**	Technology	Phase-shifting optical triangulation, twin-camera
	Camera resolution (pxl)	2M/5M
	Point scanning (mm)	0.03–0.71
	Max accuracy (mm)	0.03
	Working distance (mm)	430–1330
	Scanning area (near)/(far)	Not available
	Measurement rate (pts/s)	Not available
	Weight (kg)	5
	Dimensions (mm)	560 × 240 × 170
	Cost (k€)	20–40

**Table A2:** High-end portable scanners.

Device		
**Artec Eva**	Technology	Structured light
	Camera resolution (pxl)	1.3M
	Max resolution (mm)	0.5
	Max accuracy (mm)	0.1
	Working distance (mm)	400–1000
	Scanning area (near)/(far)	214 × 148/536 × 371
	Measurement rate (pts/s)	2M
	Weight (kg)	0.9
	Dimensions (mm)	262 × 158 × 63
	Cost (k€)	14
**Go! SCAN 3D**	Technology	Structured light
	Camera resolution (pxl)	Not available
	Max resolution (mm)	0.1
	Max accuracy (mm)	0.05
	Working distance (mm)	400
	Scanning area (near)/(far)	390 × 390/not available
	Measurement rate (pts/s)	1.5M
	Weight (kg)	1.3
	Dimensions (mm)	89 × 114 × 346
	Cost (k€)	20
**zSnapper Portable**	Technology	Structured light
	Camera resolution (pxl)	640 × 480
	Max resolution (mm)	0.3
	Max accuracy (mm)	0.05
	Working distance (mm)	350
	Scanning area (near)/(far)	Not available
	Measurement rate (pts/s)	Not available
	Weight (kg)	2.3
	Dimensions (mm)	230 × 130 × 115
	Cost (k€)	30
**Insight3**	Technology	Structured light
	Camera resolution (pxl)	1280 × 1024
	Max resolution (mm)	0.12/0.4
	Max accuracy (mm)	Not available
	Working distance (mm)	150–500
	Scanning area (near)/(far)	250 × 170/not available
	Measurement rate (pts/s)	Not available
	Weight (kg)	2.7
	Dimensions (mm)	327 × 246 × 74
	Cost (k€)	Not available

**Table A3:** Low-end portable scanners.

Device		
**Sense 2 3D Scanner**	Technology	Structured light
	Field of view (hor × ver)	45° × 57.5°
	Accuracy (mm)	1 (at 0.5 m)
	Resolution (pxl)	640 × 480
	Working distance (mm)	400–1600
	Frame rate (fps)	30
	Weight (kg)	0.59
	Dimensions (mm)	178 × 129 × 33
	Cost (€)	500
**PrimeSense Carmine 1.09**	Technology	Structured light
	Field of view (hor × ver)	57.5° × 45°
	Accuracy (mm)	1 (at 0.5 m)
	Resolution (pxl)	640 × 480
	Working distance (mm)	350–3000
	Frame rate (fps)	60
	Weight (kg)	0.22
	Dimensions (mm)	180 × 25 × 35
	Cost (€)	300
**Kinect sensor V1**	Technology	Structured light
	Field of view (hor × ver)	57° × 43°
	Accuracy (mm)	Not available
	Resolution (pxl)	320 × 240
	Working distance (mm)	400–3500 (near mode)
	Frame rate (fps)	30
	Weight (kg)	0.55
	Dimensions (mm)	279.4 × 63.5 × 38.1
	Cost (€)	100
**Kinect sensor V2**	Technology	Time of flight
	Field of view (hor × ver)	70° × 60°
	Accuracy (mm)	Not available
	Resolution (pxl)	512 × 424
	Working distance (mm)	500–4500
	Frame rate (fps)	30
	Weight (kg)	1.4
	Dimensions (mm)	249 × 66 × 67
	Cost (€)	200
**Azure kinect**	Technology	Time of flight
	Field of view (hor × ver)	120° × 120°
	Accuracy (mm)	Not available
	Resolution (pxl)	1024 × 1024
	Working distance (mm)	400–4200
	Frame rate (fps)	30
	Weight (kg)	0.44
	Dimensions (mm)	103 × 39 × 126 mm
	Cost (€)	400
**Structure sensor**	Technology	Structured light
	Field of view (hor × ver)	58° × 45°
	Accuracy (mm)	0.5 (at 0.4 m)/30 (at 3 m)
	Resolution (pxl)	640 × 480
	Working distance (mm)	400–3500
	Frame rate (fps)	30–60
	Weight (kg)	0.095
	Dimensions (mm)	119 × 28 × 29
	Cost (€)	400
**Structure sensor Mark-II**	Technology	Structured light
	Field of view (hor × ver)	59° × 46°
	Accuracy (mm)	±0.29% (Plane-fit RMS at 1 m)
	Resolution (pxl)	1280 × 960
	Working distance (mm)	300–5000
	Frame rate (fps)	54
	Weight (kg)	0.065
	Dimensions (mm)	109 × 18 × 24
	Cost (€)	500

**Table A4:** Intel RealSense depth cameras.

Device		
**RealSense SR305**	Technology	Structured light
	Depth of field of view (hor × ver × diag)	69° ± 3° × 54° ± 2°
	Data stream output resolution (pxl)	640 × 480
	RGB sensor resolution	1920 × 1080
	Working distance (mm)	200–1500
	Frame rate (fps)	60
	Weight (kg)	0.07
	Dimensions (mm)	140 × 26.1 × 12
	Cost (€)	80
**RealSense D415**	Technology	Structured light
	Depth of field of view (hor × ver × diag)	65° ± 2° × 40° ± 1° × 72° ± 2°
	Data stream output resolution (pxl)	1280 × 720
	RGB sensor resolution	1920 × 1080
	Working distance (mm)	300–10,000
	Frame rate (fps)	90
	Weight (kg)	0.072
	Dimensions (mm)	99 × 20 × 23
	Cost (€)	150
**RealSense LiDAR Camera L515**	Technology	LiDAR
	Depth of field of view (hor × ver × diag)	70° × 55° (±2°)
	Data stream output resolution (pxl)	1024 × 768
	RGB sensor resolution	1920 × 1080
	Working distance (mm)	250–9000
	Frame rate (fps)	30
	Weight (kg)	0.1
	Dimensions (diam × height) (mm)	61 mm × 26 mm
	Cost (€)	300

**Table A5:** Body scanners.

Device		
**Dimensional Imaging DI3D**	Technology	Photogrammetry
	Max resolution (pxl)	21 M
	Max accuracy (mm)	0.5
	Working distance (mm)	-
	Capture speed (ms)	-
	Dimensions (mm)	-
	Cost (k€)	20–140
**Vectra XT Scanner**	Technology	Photogrammetry
	Max resolution (pxl)	-
	Max accuracy (mm)	1.2
	Working distance (mm)	500
	Capture speed (ms)	3.5
	Dimensions (mm)	1520 × 1410 × 420
	Cost (k€)	15
**3DMDhand System**	Technology	Photogrammetry
	Max resolution (pxl)	-
	Max accuracy (mm)	0.2
	Working distance (mm)	-
	Capture speed (ms)	1.5
	Dimensions (mm)	-
	Cost (k€)	20–50
